# Comparison of psychometric methods for examining the factor structure of the Supportive Care Needs Survey- Short Form (SCNS-SF34) in Colombian cancer patients

**DOI:** 10.21500/20112084.7546

**Published:** 2025-11-19

**Authors:** Nicolás Martinez-Ramos, Gabriela Negrete-Tobar, Oscar Gamboa-Garay, Raúl Murillo

**Affiliations:** 1 Centro Javeriano de Oncología, Hospital Universitario San Ignacio. Bogotá, Colombia. Centro Javeriano de Oncología Hospital Universitario San Ignacio Bogotá Colombia; 2 Facultad de Medicina, Pontificia Universidad Javeriana, Bogotá, Colombia. Pontificia Universidad Javeriana Facultad de Medicina Pontificia Universidad Javeriana Bogotá Colombia

**Keywords:** Psychometric validation, supportive care, neoplasms, needs- assessment, Validación psicométrica, cuidados de soporte, neoplasias, evaluación de necesidades.

## Abstract

**Background:**

to ensure optimal oncology care a structured assessment of different needs is highly desirable. The Supportive Care Needs Survey in its short form (SCNS-SF34) is frequently used to such objective. The survey has been validated in several contexts; accordingly, some reports suggest alternative structures. However, there are differences in validation outcomes attributable to study heterogeneity. Thus, we aimed to test the factor structure of the SCNS-SF34 comparing different psychometric methods.

**Methods:**

an instrumental study was conducted in Bogotá-Colombia. A sample of 200 adult patients diagnosed and already treated for any type of cancer was estimated. Patients were randomly selected at a referral center; data was collected by trained personnel. To test the factor invariance, we used a parallel analysis for the exploratory factor analysis (EFA), the exploratory structural equation modelling (ESEM), and the exploratory graph analysis (EGA).

**Results:**

overall, 245 patients were recruited; 64.0% women; 83.7% lived in urban areas; 34.7% had elementary education, and all were affiliated to a health insurance company in the Colombian health system. The parallel analysis yielded 5 factors explaining 55% of variance with low goodness of fit (CFI = 0.687, TLI = 0.602, RMSEA = 0.139, SRMR = 0.040). The ESEM adjusted for 5 factors with good fit (CFI = 0.971, TLI = 0.960, RMSEA = 0.023, SRMR = 0.04), however, some items presented a Heywood effect and loaded to different domains. The EGA showed 5 node communities, but some patient care and support items were found integrated within the health systems-and-information domain. The theoretical domains showed adequate reliability.

**Conclusions:**

the 5 domains of the SCNS-SF34 showed structural validity for its application in Colombia. Further analysis in other Spanish-speaking Latin American countries are anticipated but our results suggest that ESEM and EGA approaches may be better to understand the structure of the survey.

## Resumen

**Antecedentes:**

para garantizar una atención oncológica óptima, es muy deseable una evaluación estructurada de las diferentes necesidades. La encuesta de necesidades de atención de apoyo en su forma corta (SCNS-SF34) se utiliza con frecuencia para tal objetivo. La encuesta ha sido validada en varios contextos; en consecuencia, algunos informes sugieren estructuras alternativas. Sin embargo, existen diferencias en los resultados de la validación atribuibles a la heterogeneidad del estudio. Por lo tanto, nuestro objetivo fue probar la estructura factorial de la SCNS-SF34 comparando diferentes métodos psicométricos.

**Métodos:**

se realizó un estudio instrumental en Bogotá, Colombia. Se estimó una muestra de 200 pacientes adultos diagnosticados y ya tratados por cualquier tipo de cáncer. Los pacientes fueron seleccionados aleatoriamente en un centro de referencia. Los datos fueron recogidos por personal previamente entrenado. Para probar la invariancia factorial, utilizamos un análisis paralelo para el análisis factorial exploratorio (EFA), el modelado exploratorio de ecuaciones estructurales (ESEM) y el análisis exploratorio de gráficos (EGA).

**Resultados:**

en total, se reclutaron 245 pacientes; 64,0% mujeres; 83,7% vivían en áreas urbanas; El 34,7% tenía educación primaria y todos estaban afiliados a una empresa de seguros de salud del sistema de salud colombiano. El análisis paralelo arrojó 5 factores que explican el 55% de la varianza con baja bondad de ajuste (CFI = 0,687, TLI = 0,602, RMSEA = 0,139, SRMR = 0,040). El ESEM ajustó por 5 factores con buen ajuste (CFI = 0,971, TLI = 0,960, RMSEA = 0,023, SRMR = 0,04), sin embargo, algunos ítems presentaron efecto Heywood y cargaron a diferentes dominios. El EGA mostró 5 comunidades de nodos, pero algunos ítems de atención y apoyo al paciente se encontraron integrados dentro del dominio de sistemas e información de salud. Los dominios teóricos mostraron una confiabilidad adecuada.

**Conclusiones:**

Los 5 dominios del SCNS-SF34 mostraron validez estructural para su aplicación en Colombia. Se prevén análisis adicionales en otros países latinoamericanos de habla hispana, pero nuestros resultados sugieren que los métodos ESEM y EGA pueden ser mejores para comprender la estructura de la encuesta.

## 1. Introduction

After cancer diagnosis, patients face several changes in their life given the course of the disease and its treatment. To ensure optimal care and well-being, a structured approach to assess physical, emotional, spiritual, and social needs is highly desirable (Williamson et al., 2021). Thus, a structured measure of patients' needs may help identify gaps be- tween unmet needs and healthcare delivery (Boyes et al., 2009), a situation not regularly explored (Wen & Gustafson, 2004).

One of the most frequently used instrumets to measure oncology patient needs is the “Supportive Care Needs Survey” in its long (SCNS-LF59) and short forms (SC-NS-SF34) (Bonevski et al., 2000; Boyes et al., 2009). These scales comprise 5 domains: Psychological, Health System and Information, Sexuality, Patient Care and Support, Daily Activity and Physical functionality. Particularly, the SCNS-SF34 has been adapted and validated in various contexts since its publication in 2009 (Table 1).

**Table 1:** Studies analyzing the SCNS-SF34 structure

Study	n	Cancer Diagnosis	Analysis	Factor structure
Australia (Boyes et al,. 2009)	1138	# Dx	1-) EFA, 2-) CFA 3-) Internal consistency 4-) Relation with other variables	5 factors
Japan (Okuyama et al,. 2009)	408	Breast	1-) Traducción 2-) PCA 3-) Internal consistency 4-) Relation with other variables	5 factors
China (Au et al,. 2011)	348	Breast	1-) EFA - PCA 2-) Internal consistency 3-) Relation with other variables	4 factors
Australia (Schofield et al,. 2012)	332	Prostate	1-) EFA 2-) Internal consistency 3-) Relation with other variables	5 factors
France (Brédart et al,. 2012)	384	Breast	1-) CFA 2-) Internal consistency 3-) Relation with other variables	5 factors
Germany (Lehmann et al,. 2012)	147	# Dx	1-) EFA 2-) Internal consistency 3-) Relation with other variables	5 factors
Hong Kong & Taiwan, China (Wylie et al,. 2013)	623	Colorectal	1-) CFA 2-) Internal consistency 3-) Relation with other variables	4-5 factors
Mexico (Doubova et al., 2015)	825	# Dx	1-) Translation 2-) IVC 3-) EFA 4-) Internal consistency 5-) Relation with other variables ) Test-retest	5 factors
Germany (Jansen et al., 2016)	201	Head neck	1-) CFA 2-) CFA- PCA 3-) Internal consistency ) Relation with other variables	4 factors
Mainland, China (Yuan et al., 2017)	861	# Dx	1-) Translation 2-) EFA - PCA 3-) Internal consistency ) Relation with other variables	5 factors
Turkey (Ozbayir et al., 2017)	170	Breast	1-) Translation 1-) IVC 2-) EFA -PCA 3-) Internal consistency	5 factors
Malaysia (Azman et al., 2021)	171	# Dx	1-) Translation 2-) EFA- PCA 3-) Internal consistency	5 factors
Mexico (Gálves-Hernández et al., 2021)	396	Breast	1-) Item discrimination 2-) Internal consistency 3-) EFA - PCA 4-) CFA	3 factors
Brazil (Dantas Vieira et al., 2021)	691	# Dx	1-) EFA 2-) CFA 3-) Internal consistency	4-5 factors
Portugal (Mendes-Santos et al., 2024)	336	Breast	1-) EFA - PCA 3-) Internal consistency 4-) Relation with other variables	4 factors

*Note. EFA = exploratory factor analysis, CFA = confirmatory factor analysis, PCA = principal components analysis*

Although most research has replicated the 5-domain structure, some reports suggest alternative structures, mainly due to differences in the organization of health systems and the idea that SCNS-SF34's domains are not universal (Au et al., 2011; Mendes-Santos et al., 2024). However, some differences could be attributed to the diverse techniques implemented to identify the underlying structure of the scale. Some studies used the principal components analysis (PCA), a method discouraged due to overestimating factors and its lack of coherence with psychological structural reflective models (Cunha et al., 2023). In addition, the Exploratory Factor Analysis (EFA), a widely used method, is criticized given the subjectivity and lack of standard criteria for the selection of estimation methods, extraction techniques and rotations (Goretzko, 2023; Nájera et al., 2023). Consequently, other analytical approaches have been suggested, including Exploratory Graph Analysis (EGA) and the exploratory structural equation modelling framework (ESEM).

The ESEM is a modelling technique that combines the best features of the EFA and the confirmatory factor analysis (CFA), allowing a cross-loading between items and factors, along with the goodness-of-fit. In consequence, this approach enables the estimation of error terms and the assessment of test invariance (Alamer, 2022). In recent years, this analysis has gained relevance, reflected in its various implementations for scale validation of multidimensional constructs (Henríquez et al., 2020; Martinez-Ramos et al., 2022; Mieres-Chacaltana et al., 2023; Neff et al., 2021; Trógolo et al., 2022).

On the other hand, the EGA is a network analysis focused on the estimation of direct relationships between observed indicators rather than modelling observed variables as a function of common latent traits (Golino & Epskamp, 2017). This analysis has shown in simulation studies similar or even better performance than the parallel analysis (PA), a method broadly used on the EFA's applications (Brandenburg & Papenberg, 2022; Golino, Shi, et al., 2020; Golino & Epskamp, 2017; Martínez-Ramos & Rondón Sepúlveda, 2025).

We found no theoretical or operative definition of constructs and dimensions of the SCNS-SF34, since the scale was proposed based on empirical assumptions by using only statistical criteria to define the dimensionality of the construct. However, this is not in agreement with current standards (Kyriazos & Stalikas, 2018; Muñíz & Fonseca-Pedrero, 2019); thus, we aimed to test the factor structure of the SCNS-SF34 using three different psychometric methods to better explain the SCNS-SF34 dimensionality.

## 2. Method

### 2.1 Materials and methods

An instrumental study (Montero & León, 2002) was conducted as part of the needs assessment of cancer patients at San Ignacio's University Hospital, Bogotá, Colom bia. The methods for the survey have been previously described (Negrete-Tobar et al., 2024), in which a descriptive study was conducted from May 2021 to June 2023.

### 2.2 Participants

A minimum sample size of 200 patients was estimated for the psychometric analysis. The sample size was guided by the classical guidelines of the Classical Test Theory and dimension reduction analysis (Muñíz, 2010; Osborne & Costello, 2004). The recruitment was done face-to-face in waiting rooms and chemotherapy room, or via telephone. For in-person recruitment, we randomized week-days (clusters) with a recruitment goal of ten patients per selected day; for the telephone recruitment, we selected patients in blocks of 100 from the attendance to ambulatory services during the previous week (response rate below 20%). Patients over 18 years of age with a histopathological diagnosis of neoplasia of any organ who were undergoing treatment or follow-up at the Xaverian Oncology Center were included. Patients with neurocognitive deficits or any other limitation that would prevent them from self-completing the survey were excluded.

The study was submitted to and approved by the ethics committee of the San Ignacio University Hospital (FM-CIE-044-2021) and informed consent was obtained from all in dividual participants included in the study.

### 2.3 Instruments and data collection

The SCNS-SF34 developed by Boyes et al. (Boyes et al., 2009) and translated into Spanish by Gálvez-Hernández et al. (2021) was used. This version was preferred over the Colombian version (Gutierrez & Hincapié, 2021), because the latter did not obtain any statistical validity based property. The questionnaire consists of 34 items in 5 domains: Psychological (Ps), Health systems and information (Hs), Patient care and support (Pc), Physical functionality (Pf) and Sexuality (Sx). These were developed on a five-point Likert scale (0 = not applicable; 1 = no need; 2 = low need; 3 = moderate need; 4 = high need). The scale has been shown to have adequate validity related to the internal structure and the relationship between variables, as well as optimal indicators of internal consistency, a = [0.86 - 0.96] (Boyes et al., 2009).

Before data collection, the understandability of the instrument was piloted in a group of patients different from those included in the final sample. The instrument was self-administered but accompanied by research personnel (previously trained) in the face-to-face recruitment. For the telephone recruitment a member of the research team filled out the form based on the information given by the patient.

Also an identification questionnaire was created in order to capture the patients' main sociodemographic and clinical characteristics.

### 2.4 Statistical analysis

We describe the frequency of every answer, the mean of the Likert scales with the stan dard deviation, the symmetry, the kurtosis and the homogeneity index (HI). Regarding the latter, values greater than 0.35 were considered acceptable, with adequate discriminatory capacity (Blum et al., 2013).

The 5-domain structure of the SCNS-SF-34, might be suitable for carrying out a CFA (Ala mer & Marsh, 2022); however, considering some differences between the factor structures found in previous studies (Table 1) an exploratory approach was opted. Before that, the Kaisser-Meier-Olkin (KMO) index and the Bartlett sphericity test were estimated. We used a PA based on polychoric correlations for the EFA to extract the factors. Given the sample size and the nature of the data, an Unweighted Least Squares (ULS) estimation method was used (Goretzko, 2023) with an oblique rotation assuming a moderate association between the different domains of the SCNS-SF-34 (Snyder et al., 2008).

The ESEM was also implemented via the ULS using the oblique rotation geomin. This rotation is a procedure that allows a more accurate estimate of the relations between factors and it is recommended when an exploratory objective is primarily pursued (Swami et al., 2023). The acceptance criteria for EFA was to explain at least 50% of the total variance (Streiner, 1994). The acceptance of EFA and ESEM was determined by the goodness of fit as follows: RMSEA < 0.06, CFI > 0.95, TLI > 0.95, SRMR < 0.06 (Hu & Bentler, 1999; Xia & Yang, 2019). The Cronbach's Alpha (a) and the McDonald's omega (w) were obtained as internal consistency indicators for each dimension.

The graphical regularization method with the least absolute shrinkage and selection operator (GLASSO) was used for the EGA. This penalty produces a more parsimonious graph that reflects only the most essential empirical relationships in the data; the best model was selected by the tuning parameter (X) that is chosen by minimizing the extended Bayesian information criterion (EBIC) which has been shown to accurately retrieve the simulated network structures (Golino, Moulder, et al., 2020; Golino & Epskamp, 2017; Hevey, 2018). Moreover, the Walktrap algorithm was implemented to simulate a random walk within a network, where it is assumed that the nodes of the same ad hoc community will have a greater probability of being “visited”; this intent to measure the similarity between the nodes and to group them into clusters or communities (Pons & Latapy, 2006). The Von Neumman entropy (VN entropy) and the average entropy were also estimated as fit measures, expecting low values (Golino, Moulder, et al., 2020). Despite centrality measures are commonly used to report the weightings, simulation studies have demonstrated that a node's strength centrality is roughly equivalent to fac tor loadings (Christensen & Golino, 2021). In this study, the BRM method estimated the loadings (Christensen & Golino, 2021), indicating each variable between- and within-community strength.

The R software (R Core Team, 2023) was used to perform the analysis using the packages readxl (Wickham, Hester, et al., 2023) dplyr (Wickham, Frangois, et al., 2023), psychometric (Fletcher, 2023) summarytools (Comtois, 2022), psych (Revelle, 2023), EGAnet (Golino & Christensen, 2024) and esem (Prokofieva et al., 2023).

## 3. Results

A total of 245 patients with different cancer types were recruited; 93.9% received systemic therapy; 64.0% were women; 83.7% lived in urban areas; 34.7% had finished elementary education, and 78.5% were affiliated with the Colombian health system under the contributory health insurance scheme or through private insurance. Mostly, patients were diagnosed with local tumors (58.5%), mainly located in the following organs: breast (18.30%), ovary (8.50%) multiple melanoma (7.70%) and prostate (5.30%).

All items showed HI above 0.35. Items from the dimensions Sx (Sexuality needs) and Pc (Patient Care and Support) showed a floor effect (Supplementary Table 1). Regarding the EFA, a KMO of 0.89 was found, and the Bartlett sphericity test was statistically significant (p < 0.001), indicating the adequacy of data to perform an EFA. The parallel analysis yielded 5 main factors that explained 55% of the variance; the items of the Pc domain loaded onto the Hs factor, and all communalities were above 0.30 (Table 2).

The ESEM was adjusted for 5 factors and the items Ps7 and Hs28 presented a Heywood effect (an overestimation of a given parameter). Multiple items from different domains loaded to the Ps and the Hs domains; however, unique factor loadings were observed for each item in the 5 domains (Table 2). Regarding the internal consistency, the theoretical domains showed adequate estimates (Pfa = 0.79, Pfw = 0.85, Psa = 0.90, Psw = 0.90, Sxa = 0.86, Sxw = 0.87, Pea = 0.83, Pcw = 0.88, Hsa = 0.92, Hsw = 0.93).

**Table 2:** SCNS-SF-34 EFA and ESEM results.

			EFA						ESEM		
Item	Pf	Ps	Sx	Pc	Hs	*h*2	Pf	Ps	Sx	Pc	Hs
Pf1	0.56					0.35	0,54				
Pf2	0.89					0.75	0,64	0.44			
Pf3	0.72					0.66	0,62	0.43			
Pf4	0.44					0.34	0,66			0.33	0.34
Pf5	0.62					0.47	0,75			0.32	
Ps6		0.63				0.58		0.69			
Ps7		0.78				0.75		1.01			
Ps8		0.75				0.74		0.80			
Ps9		0.81				0.58		0.56			
Ps10		0.79				0.60		0.43	0.36	-0.43	0.42
Ps11		0.76				0.63	0.37	0.37	0.47	-0.62	0.47
Ps12		0.63				0.54				-0.40	0.54
Ps13		0.60				0.57	0.37	0.31		-0.39	0.53
Ps14		0.55				0.63		0.49			0.41
Sx15			0.92			0.91		0.38	0.87		
Sx16			0.94			0.90	0.87				
Ps17		0.47				0.45	0.40	0.33	0.32	-0.35	0.32
Pc18	0.38	0.41				0.38				0.43	0.36
Pc19				0.44	0.56	0.52				0.46	0.49
Pc20					0.71	0.66	0.37				0.93
Pc21					0.75	0.67	0.31				0.88
Pc22					0.71	0.62	0.80				
Hs23					0.69	0.52	0.70				
Hs24					0.66	0.46	0.67				
Hs25					0.75	0.60	0.90				
Hs26					0.81	0.63		-0.38			0.95
Hs27					0.78	0.63	0.56	-0.59		-0.39	1.00
Hs28					0.76	0.68	0.47	-0.38		-0.40	1.03
Hs29					0.70	0.48		-0,30			0.83
Hs30					0.66	0.54	0.35				0.87
Sx31					0.78	0.65			0.80		0.30
Hs32					0.81	0.71					0.79
Hs33					0.77	0.65					0.77
Hs34					0.47	0.30					0.65

*Note. Pf = Physical functioning, Ps = Psychological, Sx = Sexuality, Pc = Pa- tient Care and Support, Hs = Health Systems and information, h'"2 = commu- nality. Factor loadings below 0.30 were suppressed.The ESEM showed better good- ness of fit than the EFA (**Table 3**); moreo- ver, the ESEM showed a lower correlation among dimensions' (x = 0.38, sd = 0.49, Min = -0.24, Max = 0.33) than the EFA (x = 0.45, sd = 0.42, Min = 0.00, Max = 0.55) (Supplementary**Table 2**).*

**Table 3:** Fit indices EFA and ESEM solutions.

Model	***X*2**	df	CFI	TLI	RMSEA	SRMR
EFA	2306.90	401	0.687	0.602	0.139	0.040
ESEM	460.524	406	0.971	0.960	0.023	0.048

**Figure 1:**
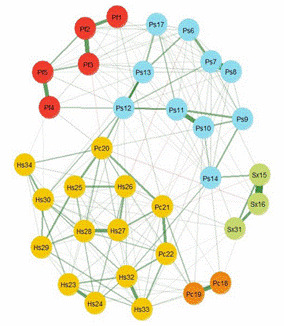
Network representation of the SCNS-SF-34. Pf = Physical functioning, Ps = Psychological, Sx = Sexuality, Pc = Patient Care and Support, Hs = Health Systems and information. The green edges reflect positive correlations, and the red edges have negative correlations between nodes. The thicker the edge the stronger the correlation. The color of the nodes indicates the communities identified.

The EGA suggested the existence of 5 node's communities (Figure 1); the network mostly comprises positive correlations; however, in the Pc domain only two nodes were identified as an independent group (Pc18-Pc19). The Ps14 is a bridge to the Sx community, and the Pc20 is a bridge with the Ps community. The network had adequate entropy indices (VN Entropy = -45.00 and average entropy = -39.68). The network loadings of the EGA are shown in Supplementary Table 3.

## 4. Discussion

This study is the first to report statistical evidence of the dimensionality of the SC- NS-SF-34 by implementing the ESEM and the EGA methods and comparing them with the EFA (PA) in a Latin-American sample of oncology patients. Despite the limitations relating to the theoretical definition of the construct, the results obtained by the three methods in the study support the 5-domains of the scale, a result similar to previous re- ports (Azman et al., 2021; Boyes et al., 2009; Brédart et al., 2012; Doubova et al., 2015; Lehmann et al., 2012; Okuyama et al., 2009; Ozbayir et al., 2017; Yuan et al., 2017).

The ESEM showed a better fit than the EFA (PA), suggesting that a non-restrictive factor model can be more accurate in explaining the underlying construct of the SC- NS-SF-34. Accordingly, some items may give information for more than one domain. For instance, Pf4 (household chores), Ps13 (Learn to feel in control of your situation) and Ps17 (Concern about the worries of those close to you), represent a physical or psychological need but simultaneously indicate a need for information about how to cope with the disease in the different contexts and how to manage the relations with their relatives.

The high correlation between physical concerns and psychological needs observed in the EGA might be explained due to similarities between physical and mental domains in oncology care (Navas et al., 2006). The strong link between Pc20 (Feedback from medical staff that the way you feel is normal) and Ps12 (Maintain a positive outlook), could be attributed to the health professionals' bias through a positive attitude on treatment out-comes (Prip et al., 2018). The proximity be- tween nodes Ps14 (Feelings about death and dying) and Sx15 (Changes in sexual sensations), could be explained by the sensation of discomfort that patients may experience while talking about those topics with their clinicians (Arroyo et al., 2022; Wang et al., 2017). Correspondingly, sexuality needs were the most central nodes (Supplementary Table 3) indicating the relevance of this topic in oncology care (Katz et al., m).

For the three models many of the Pc items (Feedback from medical staff that the way you feel is normal, Medical staff promptly attend to your physical needs, Hospital staff recognize and are sensitive to your feelings and emotional needs) loaded into the Hs dimensions. This unification has been reported previously (Dantas Vieira et al., 2021; Jansen et al., 2016), hence, the patients in our study may interpret some of the supportive care simply as information provision.

Although a CFA was not performed, given the hybrid nature of the ESEM, our results could be interpreted, as well as preliminary confirmatory evidence related to the SC- NS-SF-34 factor structure. The ESEM findings should help understand the multidimensionality of patients' needs in addition to the crosswise attribute of some needs such as informational and psychological needs, which are frequently missed even by the patients themselves (Wen & Gustafson, 2004).

Related to the internal consistency results, the Cronbach's a obtained for each dimension was similar to what has been found in psychometric studies of the SCNS-SF34, as reported in a recent meta-analysis (Lee et al., 2023). Additionally, based on the McDonald's Omega result, it can be concluded that the SCNS-SF34 exhibits an optimal reliability.

The lack of content validity and relationship with other variables is a major limitation of our study, an aspect that may clarify the lower percentage of total variance compared found by the EFA compared with other studies (Azman et al., 2021; Boyes et al., 2009; Jansen et al., 2016; Lehmann et al., 2012; Mendes-Santos et al., 2024; Ozbayir et al., 2017; Wylie et al., 2013; Yuan et al., 2017). Despite the mentioned limitations, our study contributes an evidence that could justify the usage of the SCNS-SF34 in Colombian oncology health settings.

As a suggestion for future studies, incorporating Actor-Partner Interdependence modeling into the assessment of oncology patient needs may be beneficial. In this model, patients and their support network pro- vide a set of criteria related to the oncology patient's needs (Jang & Jeong, 2021). This proposal converges with other construct assessments that imply different observers for its factor structure, such as family well-being (Martínez-Ramos et al., 2025).

## 5. Conclusions

The 5 domains of the SCNS-SF34 showed structural validity for its application in Colombia. Further analysis in other Spanish-speaking Latin American countries are anticipated but our results suggest that ESEM and EGA approaches may be better to understand the structure of the survey.

## 6. Conflict of interest

The authors report not having any conflict of interest.

## 7. Funding

This research did not receive any external funding resource.

## 8. Data availability statement

Data will be available upon request contacting the corresponding author.
